# Molybdenum Disulfide Surface Modification of Ultrafine-Grained Titanium for Enhanced Cellular Growth and Antibacterial Effect

**DOI:** 10.1038/s41598-018-28367-0

**Published:** 2018-07-02

**Authors:** Myeong Hwan Shin, Seung Mi Baek, Alexander V. Polyakov, Irina P. Semenova, Ruslan Z. Valiev, Woon-bong Hwang, Sei Kwang Hahn, Hyoung Seop Kim

**Affiliations:** 1 Department of Materials Science and Engineering, Pohang University of Science and Technology (POSTECH), Pohang, 37673 Republic of Korea; 2 Institute of Physics of Advanced Materials, Ufa State Aviation Technical University, Ufa, 450008 Russia; 3 Saint Petersburg State University, 7/9 Universitetskaya nab., St. Petersburg, 199034 Russian Federation; 4 Department of Mechanical Engineering, Pohang University of Science and Technology (POSTECH), Pohang, 37673 Republic of Korea; 5 Center for High Entropy Alloys, Pohang University of Science and Technology (POSTECH), Pohang, 37673 Republic of Korea

## Abstract

The commercially pure Ti (CP Ti) and equal-channel angular pressing (ECAP) processed Ti can contribute to the downsizing of medical devices with their superior mechanical properties and negligible toxicity. However, the ECAP-processed pure Ti has the risk of bacterial infection. Here, the coarse- and ultrafine-grained Ti substrates were surface-modified with molybdenum disulfide (MoS_2_) to improve the cell proliferation and growth with antibacterial effect for further dental applications. According to *in vitro* tests using the pre-osteoblast of MC3T3-E1 cell and a bacterial model of *Escherichia coli* (*E*. *coli*), MoS_2_ nanoflakes coated and ECAP-processed Ti substrates showed a significant increase in surface energy and singlet oxygen generation resulting in improved cell attachment and antibacterial effect. In addition, we confirmed the stability of the surface modified Ti substrates in a physiological solution and an artificial bone. Taken together, MoS_2_ modified and ECAP-processed Ti substrates might be successfully harnessed for various dental applications.

## Introduction

Metallic biomaterials have been used as medical devices to permanently replace the hard tissue of the human body such as teeth and joints where the loss is prone to occur^1^. Titanium (Ti) alloys and stainless steels have been commercialized for metallic biomaterials based on their stable mechanical properties. However, the toxicity of alloying elements has been a major problem^2–4^. Pure Ti has non-toxicity and excellent corrosion resistance, but its mechanical properties are lower than those of the commercialized materials^5–8^. In order to enhance the mechanical properties of pure Ti, ultrafine-grained (UFG) Ti with superior mechanical properties have been developed using severe plastic deformation (SPD) processes, such as high-pressure torsion (HPT)^9^ and equal-channel angular pressing (ECAP)^10,11^. In addition to the enhanced mechanical properties, there have been many studies that demonstrate an excellent biocompatibility of UFG Ti^12,13^. Furthermore, various surface treatment methods are used to achieve a biocompatible metal surface^14,15^. Among the methods, etching is the most basic surface treatment method for removing a non-uniform native surface layer and developing a uniform oxide layer with a rough surface^16^. In our previous research, we have demonstrated that the etched UFG Ti possess enhanced mechanical properties compared to the polished surface^17^.

The bacterial infections on the implant surface present a risk not only immediately after surgery but also at any time by the hematogenous or by the lymphogenous route^18–20^. Because it is impossible to completely eliminate the bacterial contamination, strategies are required for biomaterial surfaces that can prevent the initial bacterial adhesion and maintain biocompatibility. Recently, studies have been carried out on coating methods to inhibit an infection while maintaining the biocompatibility^21^. The use of hydrophilic poly(methacrylic acid) (PMMA)^22^ or protein-resistant poly(ethylene glycol) (PEG)^23^ as an anti-adhesive polymer coating on Ti surface has been studied and regarded as effective in inhibiting bacterial adhesion. However, the effectiveness of the polymer coating is only for a short period of time, and the degradation has been reported to occur after 8–12 days^23,24^.

On the other hand, two-dimensional (2D) material coatings have attracted attention based on their aqueous stability^25–29^. In a recent study, Kim *et al*.^30^ demonstrated the amount of reactive oxygen species (ROS) produced by 2D molybdenum disulfide (MoS_2_) is higher than that of graphene. MoS_2_ is a prototypical transition metal dichalcogenides (TMDs) material, and it consists of two planes of hexagonally arranged sulfur (S) atoms linked with a hexagonal plane of molybdenum (Mo) atoms. MoS_2_ is a suitable coating material for mass production process because of its simple coating method with amphiphilic behavior^31^ and low cost. Previous extensive biocompatibility study of MoS_2_ showed its low cytotoxicity and genotoxicity^32^. However, its practical application for medical implantation has rarely been reported. Also, its sulfide edge surface can introduce coupling with a peptide of the cell surface to improve cellular attachment preventing implantation problem including anticoagulation, restenosis, and thrombosis^33,34^. Despite the potential for the cellular and antibacterial behavior of MoS_2_^30,35,36^ including contact membrane stress, singlet oxygen (O^2−^) induced ROS production, the effect of MoS_2_ nanoflakes coating on the Ti surface for a medical device has not been investigated as far as the authors know.

Recently, ECAP-processed pure Ti was reported to enhance the bacterial adhesion^37^. Although the etched surface has a variety of advantages, the biocompatible surface of Ti implants can contribute to bacterial colonization and biofilm formation as well as forming a surface protein layer^38^. In the present paper, we investigated the effect of antibacterial with a model bacterium, Escherichia coli (*E*. *coli*). The cellular activities of MoS_2_ coated pure Ti with the pre-osteoblast cell were carried out *in vitro*. In addition, the ECAP-processed Ti and HF etching were used to investigate the effect of MoS_2_ coating depending on grain size and etching. Furthermore, the sustainability of the MoS_2_ coating was measured.

## Results

### Surface characterization

**Figure 1:**
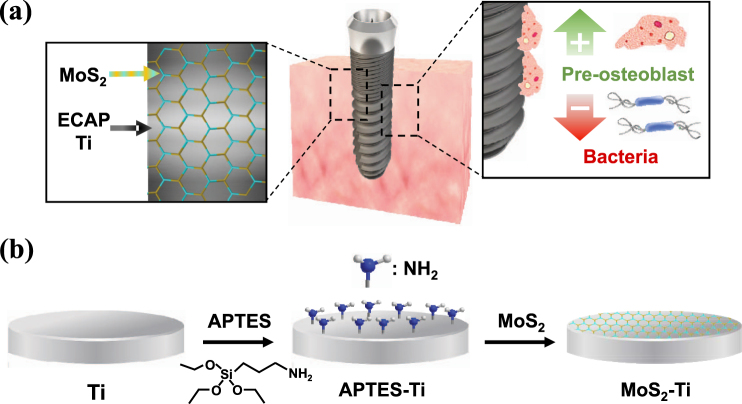
Schematics of MoS_2_ coated ECAP Ti. (**a**) Graphic illustration for mini-implant using ECAP Ti with coated MoS_2_ to improve cell proliferation and enhance antibacterial effects. (**b**) Coating MoS_2_ nanosheet with (3-aminopropyl)-triethoxysilane (APTES) on Ti surface.

**Figure 2:**
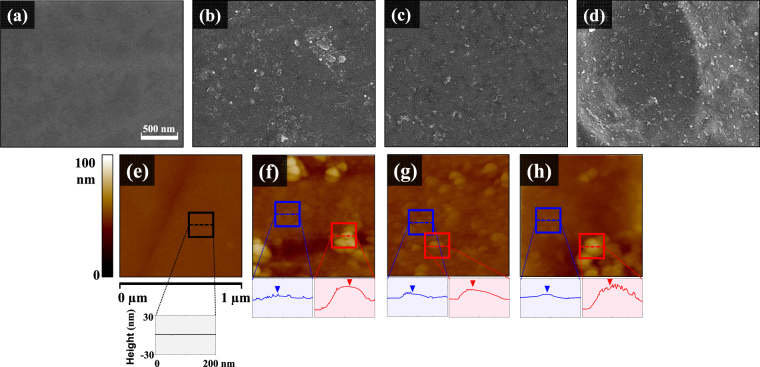
SEM micrographs and surface topographies with height profiles measured using AFM. (**a**) SEM images of CP Ti, (**b**) MS-Ti, (**c**) MS-ECAP, and (**d**) MS-eECAP surfaces. (**e**) AFM images of CP Ti; (**f**) MS-Ti; (**g**) MS-ECAP; and (**h**) MS-eECAP. The blue box represents the MoS_2_ nanoflakes section with uniform distribution, and the red box represents the cluster of MoS_2_ nanoflakes.

Figure 1 shows the schematics of MoS_2_ nanoflakes coated Ti. After the functionalization of (3-aminopropyl)-triethoxysilane (APTES) on Ti, electrostatic adhesion occurred between the positively charged Ti surface and negatively charged MoS_2_. The surface of the uncoated Ti (CP Ti) with mirror surface is shown in Fig. 2(a). The MoS_2_ nanoflakes coated surface of non-etched CG (MS-Ti) is shown in Fig. 2(b). In the case of UFG manufactured by the ECAP-Conform process, the surface as shown in Fig. 2(c,d) was obtained using the MoS_2_ coating on the non-etched surface (MS-ECAP) and the etched surface (MS-eECAP), respectively. Unlike the CP Ti, it was confirmed that the MoS_2_ nanoflakes were well distributed on the surface of the specimens. The atomic-force microscopy (AFM) results, shown in Fig. 2(e–h), are consistent with the scanning electron microscopy (SEM) results. Clusters of MoS_2_ nanoflakes of less than 50 nm were measured in each of the coated specimens.

**Figure 3:**
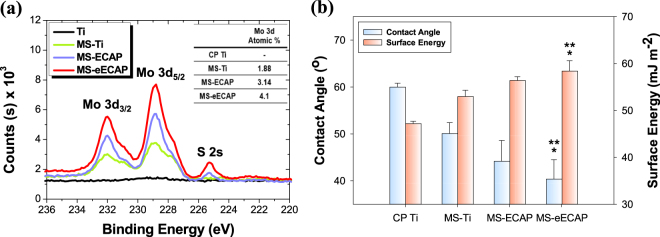
XPS spectra and wettability behavior of MoS_2_ coated specimens. (**a**) XPS spectra of Mo 3d_3/2_, Mo 3d_5/2_, and S 2 s core level peaks of the specimens. (**b**) The mean values of contact angle with calculated surface energy of each sample. ^**^p < 0.01 compared to the CP Ti substrates; and ^*^p < 0.05 compare with the MS-Ti substrate.

Figure 3(a) shows the results of X-ray photoelectron spectroscopy (XPS) to confirm the existence of MoS_2_ nanoflakes and its chemical composition. XPS results showed that Mo atomic% was higher in the order of MS-eECAP, MS-ECAP, and MS-Ti. Mo 3d_3/2_, Mo 3d_5/2_, and S 2 s peaks were not detected in the CP Ti. Wettability was examined using contact angle measurement^39,40^, and the surface energy on different surfaces are shown in Fig. 3(b). The contact angle value in the range of 48–62° and 35–50° indicate a moderately wettable and high hydrophilic surface, respectively. The surface of the MS-eECAP had a lower contact angle and higher surface energy than the CP Ti (**p < 0.01) and the MS-Ti (*p < 0.05) surfaces. Although there were no significant differences, the MS-eECAP shows improved wettability over the MS-ECAP.

Table 1 shows the roughness parameter, the mean value ± standard deviation, and the values of the surface area differences. The MS-eECAP specimen has high roughness and the surface area due to the etching process. There is no significant difference in the surface roughness and surface area differences between the CP Ti, MS-Ti, and MS-ECAP specimens without the etching process.

**Table 1:** Roughness parameter (scan area of 10 µm × 10 µm) of specimens.

	R_a_ (nm)	R_q_ (nm)	R_max_ (nm)	Surface area differences (%)
CP Ti	1.54 ± 0.37	2.51 ± 0.34	53 ± 12	0.12 ± 0.01
MS-Ti	1.87 ± 0.31	2.74 ± 0.37	82 ± 18	0.22 ± 0.04
MS-ECAP	4.04 ± 0.94	5.38 ± 1.33	71 ± 27	0.51 ± 0.06
MS-eECAP	241 ± 116*	333 ± 114*	1786 ± 655*	23.63 ± 2.32*

R_a_ is the average roughness of the absolute value of the profile height; R_q_ is the root mean square roughness of surface; R_max_ is the height of the highest peak in the roughness profile. *p < 0.05 compared to the CP Ti, the MS-Ti, and the MS-ECAP substrates.

### Cellular activities of MoS_2_ coated Ti

**Figure 4:**
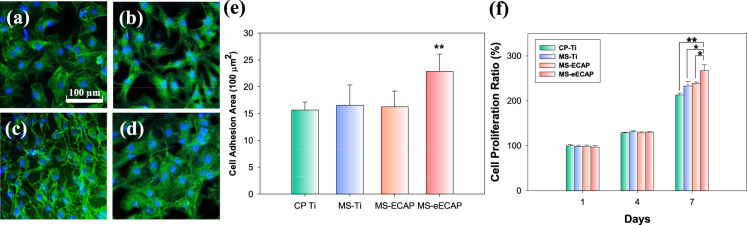
Pre-osteoblast cells spreading on the surface and proliferation rate. Fluorescence images of F-actin (green) and nucleus (blue) stained cells on the (**a**) CP Ti, (**b**) MS-Ti, (**c**) MS-ECAP, and (**d**) MS-eECAP substrates after 3 days. (**e**) The average cell adhesion area on each substrate by measuring the region of F-actin fluorescence using J software. The fluorescence images of F-actin were used false color from red to green, and merged with the nucleus fluorescence images. ^**^p < 0.01 compared to the CP Ti, the MS-Ti, and the MS-ECAP substrates. (**f**) Proliferation rate of the pre-osteoblast cells with a reference of the CP Ti substrate (^*^p < 0.05 and ^**^p < 0.01).

Confocal fluorescence imaging of F-actin (phalloidin rhodamine; pseudo green) and nucleus (DAPI; blue) stained cells on each sample were shown in Fig. 4(a–d). As shown in the figures, MS-eECAP showed much more spread and attached morphology compared with those of other samples after cell seeding for 3 days. The MS-eECAP had the higher value of the average cell adhesion area per one cell than those of the CP Ti (*p* < 0.01), MS-Ti (*p* < 0.01), and MS-ECAP (*p* < 0.01) (Fig. 4(e)). Figure 4(f) shows the cellular viability of pre-osteoblast MC3T3-E1 cells on the surface of each sample assessed after culture for 4 and 7 days. Cells on MoS_2_ nanoflake coated samples show higher cellular proliferation ratio than those on CP Ti. Interestingly, MS-eECAP sample shows the largest cell growth. It can be considered that the hydrophilic surface provides biocompatible environment compared to the hydrophobic surface.

### Antibacterial effect of MoS_2_ coated Ti

**Figure 5:**
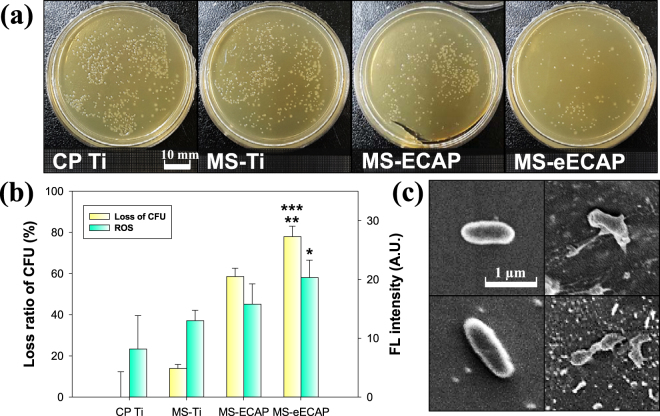
Antibacterial activity of MoS_2_ coated specimens. (**a**) Photographs of *E*. *coli* growth on bacterial culture medium (overnight incubation at 37 °C) and (**b**) bacterial growth inhibition rate of each substrate at 37 °C for 6 h. ^***^p < 0.001 and ^*^p < 0.05 compared to the CP Ti substrates; and ^**^p < 0.01 compare with the MS-Ti substrate. (**c**) SEM images of *E*. *coli* exposed to the CP Ti and the MS-eECAP substrates at 37 °C for 6 h. The left and right side images are *E*. *coli* of the surface of the CP Ti and the MS-eECAP, respectively.

To assess the antibacterial adhesion effect of MoS_2_ nanoflake coating, model bacteria, *E*. *coli*, was cultured on each sample at 37 °C for 6 h. After the incubation, attached bacteria was carefully collected and counted by measuring colony counting method (Fig. 5(a,b) shows its relative loss of viability compared to CP Ti as a control. Also, to understand the antibacterial mechanism, superoxide anion induced by ROS was measured using the singlet oxygen sensor green (SOSG) probe. The test revealed that ROS production ability of MoS_2_ on the Ti surface was effective based on the MoS_2_ coating efficiency.

Also, SEM images were obtained to observe the morphology of bacterial cell attached to each specimen. Figure 5(c) shows SEM image of the attached bacteria after 6 h incubation between CP Ti and MS-eECAP. The image shows extremely destructed bacterial morphology including holes in the cell membranes when attached to the surface of MS-eECAP. Also, cytoplasmic and intracellular component leakage were clearly observed. This observation indicates an evidence of physical damage of MoS_2_ coating on the Ti surface.

**Figure 6:**
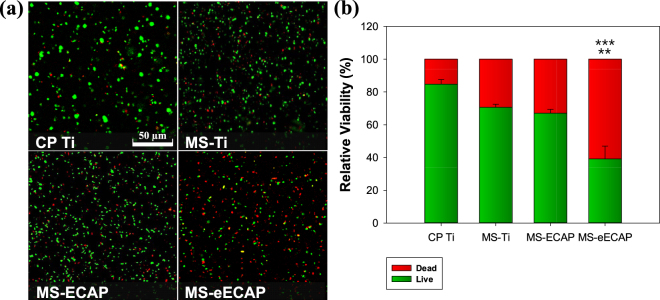
Fluorescent live-dead assay on the *E*. *coli* incubated on each substrate at 37 °C for 6 h. (**a**) Fluorescence images of damaged (Red) and viable (Green) *E*. *coli* on each specimen. (**b**) Live and dead rate of *E*. *coli* on each specimen. ^***^p < 0.001 compared to the CP Ti substrates; and ^**^p < 0.01 compare with the MS-Ti and the MS-ECAP substrate.

To further investigate the bactericidal effect, live/dead fluorescent staining was performed. Damaged or viable bacteria cells were visualized by collecting supernatant of bacterial cultured on each specimen. Figure 6(a) shows the confocal microscopy images of bacterial viability inhibition on each sample using live/dead assay with SYTO 9 (green) and propidium iodide (PI) (red) for *E*. *coli*. Obviously, the ratio of live bacteria has tendency to decrease in the following order CP Ti (84.76%, ***p < 0.001), MS-Ti (70.64%, **p < 0.01), MS-ECAP (67.03%, **p < 0.01), and MS-eECAP (39.07%). It clearly indicates the anti-bacterial effects with anti-adhesion and bactericidal effect.

### Sustainability of MoS_2_ coatings of ECAP-processed and etched Ti

**Figure 7:**
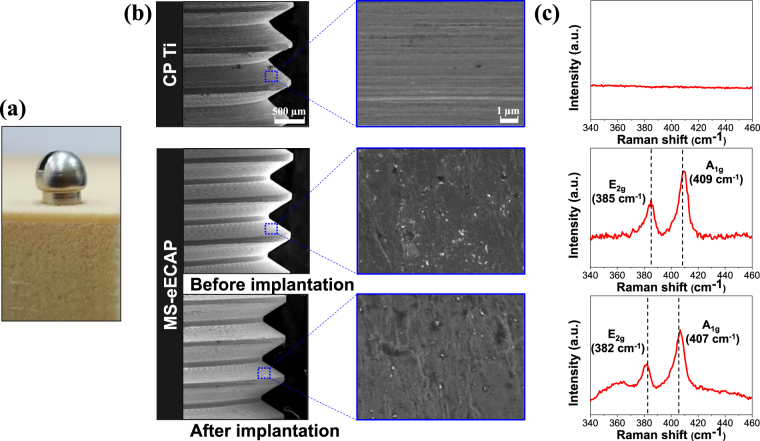
Photoimage, SEM micrographs, and Raman spectra of a prototype implant. (**a**) Photoimage showing the implantation of a prototype implant prepared with ECAP Ti to an artificial bone block. (**b**) SEM images and (**c**) Raman spectra of CP Ti and MS-eECAP screw before implantation and MS-eECAP screw after implantation from an artificial bone block.

To evaluate the persistence of the MoS_2_ coating, the immersion test in the phosphate-buffered saline (PBS) solution and the implantation test in the artificial bone block were carried out. The inductively coupled plasma (ICP) results revealed a negligible amount (less than 0.01 mg/L) of Mo in the solution immersed in each specimen in all periods. The prototype implants of MS-eECAP was inserted in the artificial bone block having similar mechanical properties with jawbone (Fig. 7(a,b) shows the surface of the CP Ti and MS-eECAP before implantation and the surface of the MS-eECAP after implantation. The remaining MoS_2_ nanoflakes on the implant surfaces were characterized using Raman spectroscopy before and after implantation as shown in Fig. 7(c). The CP Ti without MoS_2_ coating exhibited no observable peak. On the other hand, MS-eECAP showed the two most intense peaks of MoS_2_ at E_2g_^1^ (around 382–385 cm^−1^) and A_1g_ (around 407–409 cm^−1^) before and after implantation into the artificial bone.

## Discussion

It is reported that the Ti surfaces were well coated with MoS_2_ nanoflakes because the surface charge of the TiO_2_ layer is slightly negative^41^, hence the slightly negatively charged TiO_2_ surface has electrostatic interaction with groups such as positively charged -NH_3_^+^. In the same way, positively charged -NH_3_^+^ forms electrostatically adhesion with negatively charged MoS_2_. In the present study, the best coating occurred in the etched ECAP Ti. The Ti substrates have an irregular oxide distribution on the mirror surface. When the Ti surface is etched, dissolution of the Ti ion occurs with the development of rough surface, and the uniform oxide layer grows based on hydroxide formation. It is known that the oxide layer of etched Ti is thicker than the unetched surfaces, and during etching O atomic% increases and more hydroxyl group is distributed on the surface^42,43^. This acidic treatment can result in efficient APTES functionalization on the surface. Consequently, the MoS_2_ coating was well formed in the etched ECAP Ti due to the largest surface area and surface charge interaction. The ECAP Ti showed more MoS_2_ coating than the CG-Ti because the nanocrystalline Ti is reported to have a higher amount of dense oxide layer than CG Ti^44^. The active external amine groups derived from the APTES treated nanocrystalline TiO_2_ layer allowed the MoS_2_ coating to work well.

Recently, a kind of TMDs is reported to have highly conducting and hydrophilic property^45,46^. Also, MoS_2_ is known to have amphiphilic properties^31^. Hence, MoS_2_ can have hydrophilicity and polarity because it can absorb water molecules more easily through a hydrogen bond, for example, Mo-S **· · ·** H-O-H^47^. In addition, the Ti substrates can also absorb water molecules because of the OH ions from the H_2_O or the hydroxyl group in the Ti oxide layer. Thus, the MoS_2_ coated Ti substrates will have a more hydrophilic surface than the uncoated CP Ti. For this reason, MS-eECAP surface with the larger surface area and more MoS_2_ coating showed the highest wettability.

The role of the surface in metallic biomaterials is very important because it can directly affect the cellular responses mediated by the extracellular matrix (ECM) proteins^14,15^. Especially, hydrophilic surfaces have an important role in the adsorption of protein and cell activity. Cell adhesion occurs through the ECM proteins and integrin cell membrane receptor on the metallic surface. The ECM proteins such as fibronectin and fibrinogen bind the integrin to support cell adhesion and trigger cell proliferation and differentiation^15^. Hydrophilic surfaces dominate the cell-substrate adhesion because of the well-absorbed effect of the ECM protein, and the cells have a more spread shape^14,48^. Additionally, the higher wettability of the UFG Ti substrates contributes by improving the adsorption of proteins, cell adhesion, proliferation, and differentiation^49^. The wettability results and trends are well matched in osteoblast cell proliferation test. The cells on MS-ECAP are well spread and attached to the surface due its high hydrophilicity and surface energy. Our results showed that the etched and MoS_2_-coated ECAP Ti substrate, which has a rough and hydrophilic surface (contact angle = 40.4 ± 4.1°) resulted in better adhesion and proliferation of pre-osteoblast cells than those of the other specimens.

Another major challenge is the prevention of bacterial infection after implantation of biomaterials. The infection can cause tissue or bone loss around the implant, leading to an additional treatment or removal of the implant. Therefore, efforts are required to reduce the surface infection. Graphene is one of the typical 2D layered materials with good biocompatibility and antibacterial effect^27–29^. In case of antibacterial effect, it has been reported that the effect of graphene is due to the induction of physical damage to the surface of the bacteria and its oxidative stress, which could disrupt microbial viability. Interestingly, in a recent study, the amount of ROS produced by MoS_2_ is higher than that of graphene^30^. Also, MoS_2_ provides the possibility of potential 2D material surfaced coating for the antibacterial surface.

In the present study, we observed that the MoS_2_ nanoflakes coated on the Ti surfaces induce cytoplasmic and intracellular component leakage, followed by physical damage. In addition to the physical damage to the cellular membrane, the chemical damage including singlet oxygen-mediated stress is another important issue for bactericidal effect. The SOSG assay showed significant singlet oxygen generations among MoS_2_ coating conditions. Herein, CP Ti did not produce any singlet oxygen species and set to be the control. MS-eECAP shows much higher singlet oxygen generation than the other specimens following to MoS_2_ coating efficiency. This trend is well matched to the previously reported antibacterial activity of MoS_2_. Here, MoS_2_ coated surface showed both antibacterial attachment and bactericidal effect. It is considered from two aspects. One is the physical aspects such as blade-like edge and the other is the chemical aspects including singlet oxygen and negative charge retraction from MoS_2_^30,35^. Also, the MoS_2_ surface coating may promote charge transport from the cell membrane to the nanoflakes due to charge transfer mechanism. This results in the destruction of the cellular membrane through oxidation lead to cell death^50^.

Live/dead assay was performed on each surfactant of the specimen for quantitative analysis of dead cells. The SYTO 9 and PI stains differ both in their spectral characteristics and in their ability to penetrate the healthy bacterial cell membrane. When used alone, the SYTO 9 stain generally labels all bacteria with both intact and damaged membranes. In contrast, PI penetrates only bacteria with damaged membranes, causing a reduction in the SYTO 9 stain fluorescence when both dyes are present^51^. Thus, with an appropriate mixture of the SYTO 9 and PI stains, bacteria with intact cell membranes are stained with fluorescent green, whereas bacteria with damaged membranes are stained with fluorescent red. Based on this experiment, due to the physical and chemical aspects of the MoS_2_, cytoplasmic and intracellular component leakage occurred and the destruction of the cellular membranes was stained with red. Thus, the MoS_2_ coating on the Ti surface prevents the formation of bacterial colonization and biofilm. In particular, the bacteria-killing effect increased in proportion to the MoS_2_ coating efficiency.

There already exist different anti-adhesive polymer coating methods for Ti. However, the polymer coating is prone to degradation in aqueous environments after 8–12 days^23^. Thus, the polymer coating has a major disadvantage in terms of long-term stability^24^. On the other hand, it is well known that 2D materials are stable in aqueous environments^25,26^. Our results show that the MoS_2_ nanoflakes coated on the Ti surface are completely retained for one month in the PBS solution. In addition to the stability in the solution, the MoS_2_ coating exhibits good wear resistance^52^. For this reason, the MoS_2_ nanoflake coating layer remained intact after insertion of the prototype implant into the artificial bone block. These results show that MoS_2_ nanoflake coating has an advantage in long-term stability.

Based on the work by Truong *et al*.^37^, the ECAP-processed pure Ti has a risk of bacterial adhesion. However, the MoS_2_ coating on the ECAP-processed Ti shows more antibacterial effect with enhanced cellular activity than the polished pure Ti surfaces. Furthermore, the MoS_2_ coating on the ECAP-processed and etched Ti has the most sustainable biocompatible and antibacterial surface. Therefore, we propose MoS_2_ coating as a practical application for Ti medical devices.

## Conclusions

The MoS_2_ nanoflakes were successfully coated on the pure Ti substrates through electrostatic interaction. The MoS_2_ nanoflakes were coated best on the MS-eECAP substrates with the largest surface area and both negative surface charge and plentiful hydroxyl groups, followed by the MS-ECAP and MS-Ti. The contact angle measurement and cellular response results showed that the MoS_2_ coated Ti substrates had more hydrophilic and biocompatible surface than the uncoated Ti. The bacterial cytotoxicity is related to the ROS production. Therefore, the MoS_2_ nanoflakes can induce membrane stress by damaging or disrupting bacterial membrane, and lead to bacteria death. Finally, the MoS_2_ coated Ti substrates showed more antibacterial activities than the uncoated Ti. As results of ion release analysis in PBS solution and implantation test in the artificial bone block, the MoS_2_ coating was well sustained on the surface of the MS-eECAP substrates. The findings of this study will shed light on the new coating method of medical devices aimed at antibacterial coating using 2D materials, especially MoS_2_.

## Materials and Methods

### Materials and surface treatment

Commercially pure Ti (CP Ti Grade 4, Dynamet company) rods were used for investigations. Processing of UFG Ti rods with 12 mm in diameter was carried out via ECAP-Conform and drawing. The 8-pass ECAP-Conform process was performed at 200 °C, followed by drawing to obtain a rod with a diameter of 6.7 mm. In our previous research, the grain size of the specimens after the SPD process was reduced from 25 μm (CG) to 200 ± 30 nm (UFG)^10^.

For surface and bio characterization, the disks were prepared with a dimension of 6 mm diameter and 1 mm thickness. The specimens were polished down to 0.25 μm using diamond suspension and subsequently polished with 0.05 μm water free colloidal silica to achieve a mirror surface. The mixture of nitric acid and hydrofluoric acid solution, 30 mL HNO_3_ (30-vol%) + 3 mL HF (3-vol%) + 67 mL H_2_O, was used to etch the specimens for 20 minutes at room temperature. After etching, the etched specimens were washed with deionized (DI) water then for 10 s in 99.9 vol% ethanol in an ultrasonic bath.

### Preparation of MoS_2_ nanoflakes

Each sample was immersed in 2% APTES (Sigma-Aldrich) ethanol solution. Then, MoS_2_ pristine nanoflakes solution (Graphene Supermarket Inc.) was poured on the positively charged samples. After an hour, all samples were dried using an air gun. This MoS_2_ coating process was repeated 3 times to introduce a few layer of MoS_2_ on the surface.

### Surface characterization

The surface topographies were observed using field emission scanning electron microscopy (XL30S FEG, Philips electron optics B.V.) with accelerating voltage of 5 kV. Surface topographies and roughness parameters were measured using AFM (Veeco Dimension 3100 and Nanoscope V7.0, Veeco). The surface topographies were performed at nano-scale with a scan size of 1 × 1 μm^2^ considering the size of the MoS_2_ nanoflakes and bacteria. The roughness parameters were examined at nano- and micro-scale with a scan size 10 × 10 μm^2^. The tapping mode was used and the scan rate was set to 1.2 and 0.6 Hz for 1 × 1 and 10 × 10 μm^2^ scan size, respectively. Four specimens from each group were examined to evaluate the average roughness parameters.

The elemental composition was evaluated using XPS (ESCA LAB250, VG scientific), and the monochromatic Al Kα source was used at 15 kV (500 μm spot size). The analyzed area of each specimen was 1.1 × 1.1 mm^2^, and spectra were quantified and analyzed automatically by the manufacturer’s software.

Wettability was measured using contact angle measurement (Smartdrop, Femtofab). 5 μL of deionized water was dropped onto each specimen with an autopipette (Finnpipette Novus, Thermo Scientific) at 25 °C. The average contact angle was measured by the sessile drop method in four specimens of each group. The solid surface energy was calculated using the Young’s equation^39^ as follows:

1 $$\cos \,\theta =-\,1+2\sqrt{\frac{{r}_{sv}}{{r}_{lv}}}{e}^{-\beta {({r}_{lv}-{r}_{sv})}^{2}},$$

where $\theta$, ${r}_{{sv}}$, and *β* are the advancing contact angle (radian), the solid surface free energy, and the constant value (0.0001247 m^2^/mJ), respectively. ${r}_{{lv}}$ is the liquid surface tension between deionized water and air (72.0 mJ m^−2^ at 25 °C)^40^.

### Cellular characterization

Osteoblast precursor cells, MC3T3-E1 (Korean Cell Line Bank), were cultured in alpha minimum essential medium (α-MEM, WelGENE Inc.) with 10% fetal bovine serum (FBS, Gibco), 100 U mL^−1^ penicillin (Gibco), and 100 µg mL^−1^ streptomycin (Gibco) at 37 °C in a humidified atmosphere of 5% CO_2_. Before the cell culture, all the specimens were sterilized by immersing in 70% ethanol for 10 min. After drying under UV lamp in the clean bench with continuous air flow for 2 h, MC3T3-E1 cells at a density of 2 × 10^3^ mL^−1^ were cultured on each sample for 24 h in 48-well plate. After a day, each sample was transferred to a new 48-well plate to eliminate the cells attached to the surface of the culture plate, not to that of the specimen. The cultured cells were assayed with cell counting kit-8 (CCK-8, Dojindo molecular technologies) solution. The plate was incubated for 1 h after treated by CCK-8 solution and measured at 450 nm with a microplate reader (EMax microplate reader, Bucher Biotec AG, Basel) for 1, 4, and 7 days after seeding. Four specimens were taken from each group to derive an average value, and the control group was the uncoated CG Ti substrate.

Cells were fixed with 4% formaldehyde and PBS (Gibco) solution to evaluate cellular morphology by confocal fluorescence microscopy. Then, the cells were permeabilized with cold acetone, washed three times with PBS solution, and incubated with 4% Texas red phalloidin (Sigma-Aldrich) diluted in PBS solution for 30 min. After incubation and additional washing, cells were mounted with DAPI staining mounting gel (Vector Laboratories Inc., Burlingame). Fluorescence images were observed using confocal microscopy (Leica TCS-SP5-MP-SMD, Leica Microsystems Wetzlar). The area of cell adhesion was measured using the ImageJ software (Sun Microsystems Inc.). Ten cells from each specimen were examined to evaluate the average area per one cell.

### Microbial activity analysis

*E*. *coli* K-12 strain of DH5a (the Korean Culture Center of Microorganisms) was cultured in LB broth (Thermo Fisher Scientific, Waltham, MA) at 37 °C overnight before testing. Bacterial cells were then harvested by centrifugation and washed 3 times in PBS. After that, cells are diluted to 5 × 10^5^ CFU mL^−1^ in PBS. 500 μL of the bacterial solution was spread to each sample in 48-well plate and incubated for 6 h at 37 °C. The supernatant of each sample was eliminated carefully and washed three times by pipetting with 1 mL of PBS to collect attached bacterial cells on the surface. 30 ${\rm{\mu }}{\rm{L}}$ of washing solution was spread to prepared LB agar plates and incubated for overnight. Bacterial cells dispensed on the CP Ti surface are regarded as a control. The colonies were counted to estimate the attached viable bacterial cells on the surface of each sample. Relative viability was calculated by the following formula: Loss of viability (%) = (counts of control − counts of sample incubated with each experimental condition)/counts of control × 100 (%).

The generation of singlet oxygen (^1^O_2_) was determined using the SOSG reagent (Invitrogen Co.). Each sample was incubated with bacterial cells with the same method as mentioned above. After 6 h, prepared SOSG solution was added to each sample to a final concentration of 15 μM. 100 μL of each supernatant is transferred to 96-well plate and absorbance measured by microplate reader at 540 nm. Bacterial cells dispensed on an empty well served as a control and each absorbance was normalized to that of control.

Live/dead staining of bacteria was performed using commercial bacterial viability kits (Invitrogen Co.). 6 mM SYTO 9 stain and 30 mM PI mixture was dissolved to DI water. Each sample was incubated with bacteria in the same condition as mentioned above to find out the ratio of dead or living bacteria in the supernatant (depending on MoS_2_ coating condition). After 6 h, 5 μL of each supernatant and prepared staining mixture was trapped between a slide and 18 mm square coverslip using immersion oil. Confocal microscopy image was obtained at 480/500 nm filter for green stained SYTO 9 and 490/635 nm for red stained PI. The number of bacteria stained with each dye was counted and represented as the average of the 4 highest areas.

### Persistence test

The elemental analysis was measured using inductively coupled plasma atomic emission spectroscopy (ICP-AES, Iris advantage, Thermo Elemental). After MoS_2_ coated specimens were immersed and incubated in PBS solution at 37 °C, the amount of Mo in solution was measured after one week, two weeks, and one month later.

A prototype dental implant with a screw diameter of 3.8 mm was prepared using the CP Ti and ECAP-processed Ti. After polishing the CP Ti and ECAP-processed Ti, the ECAP-processed Ti screw was etched and coated with MoS_2_ nanoflakes. Then, the control and the coated screw were implanted into an artificial bone block provided by Dio Implant Co. using an electrical motor drill. The stability of MoS_2_ nanoflake coating on a screw-shaped etched ECAP Ti was analyzed using SEM and Raman spectroscopy (Alpha 300 R, WITECH) before and after implantation. Raman spectra were obtained with 532 nm Nd:YAG laser by keeping its power up to 5 mW.

### Statistical methods

All data were assessed by the analysis of variance (ANOVA), and Student’s *t*-test was performed for evaluations between groups. The significance level was set at *p < 0.05, **p < 0.01, and ***p < 0.001.
